# Direct Laser Interference Patterning (DLIP) on PEEK Coating for Biomedical and Dental Applications

**DOI:** 10.1055/s-0045-1812108

**Published:** 2025-10-22

**Authors:** Muhammad Amber Fareed, Khalil Ahmad

**Affiliations:** 1 Department of Clinical Sciences, College of Dentistry, Ajman University, Ajman, United Arab Emirates; 2 Centre of Medical and Bio-Allied Health Sciences Research, Ajman University, Ajman, United Arab Emirates; 3 Department of Materials Science & Engineering, Institute of Space Technology Islamabad, Islamabad, Pakistan

**Keywords:** electrophoretic deposition, dental implants, DLIP, antibacterial activity, *E. coli*, surface roughness

## Abstract

**Objective:**

Polyether ether ketone (PEEK) is widely recognized for its biocompatibility and mechanical stability, making it a promising coating material for dental implants. However, unmodified surfaces may lack optimal properties for osseointegration and antibacterial resistance. Direct laser interference patterning (DLIP) is an advanced technique for introducing controlled micro- and nanoscale surface features to enhance implant performance. This study aims to investigate the surface characteristics, antibacterial effect, and biocompatibility of DLIP-functionalized PEEK coatings electrophoretically deposited on 316L stainless steel (SS).

**Materials and Methods:**

PEEK was deposited onto 316L SS substrates via electrophoretic deposition and subsequently functionalized using DLIP to create periodic surface patterns with spatial periods of 1, 1.5, and 2 µm. The modified surfaces were characterized using scanning electron microscopy, contact angle (wettability), and surface roughness measurements. Antibacterial activity was assessed using the turbidity method against *Escherichia coli* . Biocompatibility was evaluated via MG-63 osteoblast-like cell viability analysis.

**Results:**

The DLIP-functionalized PEEK surface with a 1.5-µm spatial period exhibited the most favorable surface features, with a contact angle of 92 ± 1° and surface roughness of 2.04 ± 0.03 µm. This configuration significantly inhibited *E. coli* growth and achieved 80% cell viability, indicating enhanced antibacterial properties and biocompatibility.

**Conclusion:**

DLIP is an effective technique for functionalizing PEEK coatings, improving key surface characteristics that support antibacterial activity and osteoblast cell compatibility. Among the tested configurations, a 1.5-µm spatial period yielded the most promising results.

**Clinical Relevance:**

This study supports the application of DLIP-functionalized PEEK coatings for dental implants, offering a novel and translatable surface.

## Introduction

Titanium (Ti) and Ti-alloys-based dental implants are regarded as the gold standard due to their physical properties, mechanical properties, and biocompatibility, and are recognized as similar to natural bone. However, dental and maxillofacial implants can be made from various biocompatible materials, including polyether-ether-ketone (PEEK) and polyether ketone, stainless steel (SS), carbon, platinum, titanium, cobalt-chrome alloys, magnesium, and zirconia. 1 2 3

Bioactive surface modifications significantly enhance the osseointegration and longevity of dental implants. 1 4 The osseointegration involves the direct bonding of bone to the implant surface, which can be facilitated by the properties of PEEK, ensuring stability during functional loading. PEEK can be used as a coating on implants to enhance their compatibility with bone and promote osseointegration, allowing for better integration with the jawbone. Coatings like PEEK can help improve the dynamic interaction at the tissue-implant interface, contributing to the overall success of the implant. 5 6

Biomedical and maxillofacial implants are made of specific metals and their alloys, including titanium and its alloys (Ti–6Al–4 V), cobalt-chromium (Co-Cr-Mo) alloys, and 316 low-carbon SS (316L SS) are commonly utilized as bone fixators in knee and hip prostheses, orthodontic wires, dental implants, and external fixators. 7 8 Among these materials, SS 316L is particularly favored due to its excellent mechanical strength, ductility, high fracture toughness, and cost-effectiveness. 8 9 However, in the presence of bodily fluids, SS 316L implants are prone to pitting corrosion. 7

These metals exhibit load-bearing ability due to their good mechanical strength, wear, and corrosion resistance. The implants of titanium and its alloys and Co-Cr-Mo alloy are costly due to their processing cost as compared with 316L SS implants, which are cost-effective. However, 316L SS implants are poor in biocompatibility and corrosion resistance as compared with titanium and cobalt-based implants. Due to damage to the protective oxide layer on the 316L SS implants, there is a chance of uncontrolled leaching of toxic ions from the implant surface causing different biological problems in the human body. 10 There is a chance of bacterial growth such as Gram-positive ( *Staphylococcus aureus* ) and Gram-negative ( *Escherichia coli* ) on the surface of the implants in the human body, which leads to the formation of biofilm. 10 11 12 13 This biofilm also causes allergic infection, etching, and other medical traumas. Both factors are responsible for the failure of an implant and necessitating its removal from its site and causes of reversionary surgery. 11 14 Before implantation, some precautionary steps against these bacterial infections are taken. The surface of the 316L SS implant is modified by depositing various kinds of biocompatible, bioactive, bioinert, wear, and corrosion resistance coatings to minimize the risk of failure of an implant. 15 16 17

Surface modification of 316L SS implants can be performed via electrophoretic deposition (EPD), which is a facile technique to deposit different kinds of polymers, bioactive glass nanoparticles, and natural herbs. 2 PEEK is a versatile synthetic biopolymer whose elastic modulus (3–5 GPa) is near to that of the cortical bone (7–30 GPa). 3 PEEK has been used as a coating material on 316L SS implants via EPD to improve its wear and corrosion properties. Earlier studies demonstrated that different kinds of PEEK-based antibacterial and corrosion-resistance coatings were deposited on 316L SS, which inhibited the growth of bacteria and improved the corrosion resistance. 18 19 20 21 22

The coating improves specific properties like biocompatibility, bioactivity, bioinertness, wear, and corrosion properties of metallic implants like 316L SS, titanium, and cobalt-chromium-molybdenum alloys used for biomedical applications. These properties can be further improved by functionalizing the coating surface through the direct laser interference patterning (DLIP) technique, which is a robust technique for texturing of coating on the implant or surface of an implant. DLIP technique is based on the local surface functionalization process, which creates periodic surface patterns with adjustable pitch and geometry when two or more coherent laser beams interfere with the surface of a material. 23 24 Micro- and submicron-periodical structures of a surface play a vital role in modifying its properties. 25 26

DLIP is a versatile technique that also improves the biological properties of the surface. DLIP creates micro- and nanostructures on the surface, which significantly reduces the adhesion of bacteria and causes biofilm formation by reducing the contact area for bacterial adhesion, or it produces such features on the surface that are too small or too sharp that such surface does not support the bacteria to colonize on that surface. Similarly, DLIP modifies the surface in such a way that the functionalized surface acts like a physical trap for bacteria, where these cannot move and grow, and in some cases, the DLIP functionalized surface disrupts the aggregate of bacteria and prevents the biofilm formation. 27 28 29 DLIP technique is a very useful technique that functionalizes the surface of an implant.

In the present study, we deposited PEEK coating on 316L SS via EPD at a potential of 25 V for 3 minutes as deposition time and maintained the interelectrode distance during PEEK deposition at 5 mm. We performed the DLIP technique, which functionalized the surface of PEEK coating and inhibited bacterial growth and minimizing the risk of biofilm formation on the surface of PEEK coating for biomedical applications. DLIP technique is a very useful technique that functionalizes the surface of an implant and also the surface of coating deposited on either 316L SS, titanium, and its alloys, cobalt-chromium-molybdenum alloy used for biomedical applications.

## Materials and Methods

### Two-Beam Direct Laser Interference Patterning

PEEK coating was deposited on 316L SS via EPD at an applied potential of 25 V for 3 minutes as deposition time and subjected to the DLIP process. PEEK coating parameters used in this study were consistent with those parameters that were reported in our previous study. 30 A pulsed laser (IS400–1-G Nd: YAG, Edgewave, Wurselen, Germany) with a wavelength of 1064 nm and a pulse duration of 10 PS was used. A two-beam DLIP configuration was employed, in which the laser beam from the laser source was directed into the DLIP head by means of multiple mirrors. A diffractive optical element configuration then divided the beam into two coherent subbeams. A prism placed on a positioning stage was used to align these coherent beams in a parallel fashion. The distance between the subbeams and, ultimately, the spatial period may be changed by adjusting the prism's position. Lastly, a lens at the bottom of the DLIP head with an intercepting angle *θ* overlapped the beams onto the surface of the PEEK coating. 31 Two-beam DLIP with 10 PS pulsed laser created periodic patterns with different spatial periods of 1, 1.5, and 2 µm on the area (1 × 1) cm ^2^ of samples of PEEK coating deposited on 316L SS via EPD. These PEEK coating samples were cleaned with ethanol before DLIP processing. DLIP unfunctionalized PEEK coating, which has no separation between its ridges, was considered as sample (S0), while DLIP functionalized PEEK coating samples having periodic patterns with spatial periods of 1, 1.5, and 2 µm were considered as samples (S1), (S1.5), and (S2), respectively, for conciseness and clarity in this study. 32 Fig. 1 shows the DLIP configuration employed on PEEK coating deposited on 316L SS via EPD to create periodic patterns with spatial periods of 1, 1.5, and 2 µm.

**Fig. 1:**
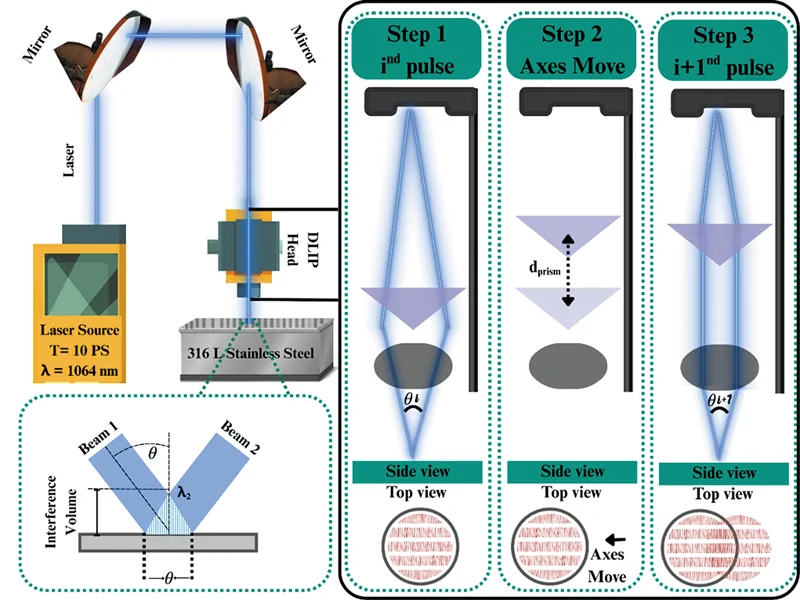
Schematic representation of the direct laser interference patterning (DLIP) optical configuration at different steps used in the laser experiments.

### Characterization of DLIP Fabricated on PEEK Coating

#### Topographical Analysis

Microstructure and topography of S1, S1.5, and S2 were observed by field emission scanning electron microscope (SEM; LEO 435 V P, Carl Zeiss AG, Germany), which was equipped with energy dispersive X-ray spectroscopy. These samples were sputtered by Q150/S (Quorum Technologies) with a thin layer of approximately 5 nm of gold and palladium, which made the surface conductive to avoid the charging artifacts. During SEM analysis of these samples, an accelerating voltage of 5.00 kV with a working distance of approximately 4 to 10 mm was used. SEM images were taken at various magnifications of (5.00–25 KX) by a secondary electrons detector to characterize the surface morphology of S1, S1.5, and S2 samples. 19

#### Contact Angle

The measurement of contact angle of a surface by ImageJ software is an acceptable technique, which provides almost the same fundamental measurement as a goniometer. The values measured by ImageJ software are accepted and have been published in many studies. But in future we will consider it an important point while measuring the contact angle of any surface, as this method has been adopted by various researchers. The sessile water droplet method was used to measure the contact angle of S0, S1, S1.5, and S2 to determine the wettability response for cellular response, their growth, and proliferation for regeneration of bone. The wettability response of S0, S1, S1.5, and S2 was determined by measuring the contact angle values of these surfaces. Earlier studies reported that contact angle values in the range of 35 to 80 degrees of the surface of any surface are a favorable surface for cellular response. The surface should be neither hydrophilic nor hydrophobic as it is favorable for cellular response. 33 A deionized water droplet of 5 µL was dispensed on the surfaces of S0, S1, S1.5, and S2, and digital images were taken with a digital camera. These images were imported into the ImageJ software to measure the values of the contact angle of each sample. Each experiment was repeated three times and the average value of surface roughness with standard deviation was reported. 19

#### Surface Roughness

The average surface roughness of S0, S1, S1.5, and S2 was evaluated by a profilometer (TMR 360). 19 A diamond-tipped stylus of the profilometer moved 10 mm in reciprocating mode on the S0, S1, S1.5, and S2 surfaces and recorded the data then, and the inbuilt software in the profilometer calculated the values of average surface roughness. The experiments were performed three times, and the average data was reported with standard deviations. 30

#### Antibacterial Analysis

Antibacterial effect of S0, S1, S1.5, and S2 was evaluated by the turbidity test. The triplicate of each sample was subjected to a turbidity test. Each sample was immersed in 1 mL of Luria Broth (LB) media poured in wells of 24-well plates. The LB media of 1 mL was inoculated with 20 µL of cultured Gram-negative bacteria ( *E. coli* ), and its optical density (OD) was measured at 600 nm, and it was fixed in the range of 0.015 to 0.017. These samples were immersed in these wells and incubated at 37°C for 24 hours. OD at 600 nm (OD _600_ ) of each sample was checked by ultraviolet-visible (UV-Vis) spectrophotometer (Genesys, 10S UV–Vis) after 1, 2, 3, 4, 6, and 24 hours. The results were reported with standard deviation for the validity of the data.

#### Cell Viability

The viability of human osteosarcoma (MG-63) cell line against S0, S1, S1.5, and S2 was checked by water soluble tetrazolium (WST-8) assay as detailed in Nawaz et al. 34 Briefly, cryopreserved MG-63 were thawed and diluted in 20 mL of culture medium in a canted neck flask at 37°C and 5 % CO _2_ for 48 hours. The culture medium comprised of 89, 10, and 1% of Dulbecco's modified Eagle medium, fetal bovine serum, and penicillin/streptomycin, respectively. After 48 hours, the culture medium was removed and adherent MG-63 cells were collected via trypsinization for 4 to 6 minutes followed by centrifugation at 800 relative centrifugal force. The collected cell pellet was diluted in fresh culture medium followed by counting them per mL using hemocytometer. A few 5 × 10 ^4^ cells were introduced to different wells of well plate except the first three wells with culture medium only referred to as blank. Next, three wells were filled with 5 × 10 ^4^ cells only referred to as tissue culture plate (TCP). S0, S1, S1.5, and S2 were introduced in the next well in triplicate. The well plate was then incubated again at prestated conditions for 48 hours. Afterwards, 100 µL from each well was transferred to 96-well plate in similar order and the WST-8 dye (10 µL) was added to each well. The 96-well plate was then incubated back for 2 hours followed by its absorbance measurement at 450 nm through enzyme-linked immunosorbent assay microplate reader (Accuris-9600). The absorbance value of TCP was considered as 100% and rest of values were reported in percentage by comparing it with TCP.



## Results

### Topographical Analysis

SEM images of S1, S1.5, and S2 were analyzed to get insight into the topography after DLIP, which functionalized the surfaces of the PEEK coating deposited on 316L SS. SEM image in Fig. 2 A at a magnification of 10 KX revealed that a pattern was created on the PEEK coating by DLIP. SEM image of Fig. 2B at higher magnification approximately 25 KX revealed the DLIP-functionalized finer structure of S1. The patterns showed that the ridges were straight but irregular due to partially removed PEEK coating from their tops, resulting in a nonuniform coating surface with a consistent separation of 1 µm between adjacent ridges of S1. Similarly, the subsequent SEM of Fig. 2C of S1.5 and the SEM image of Fig. 2E of S2 at magnification 10 KX also revealed the same topography of ridges created by DLIP in the SEM image ( Fig. 2A ). The SEM images of Fig. 2D of S1.5 and the SEM image of Fig. 2F of S2 at high magnification (25 KX) also showed the irregular and nonsmooth shaped ridges except the spatial period between their ridges was different, which was 1.5 and 2.0 µm, respectively, as clearly highlighted in SEM images shown in Fig. 2 .

**Fig. 2:**
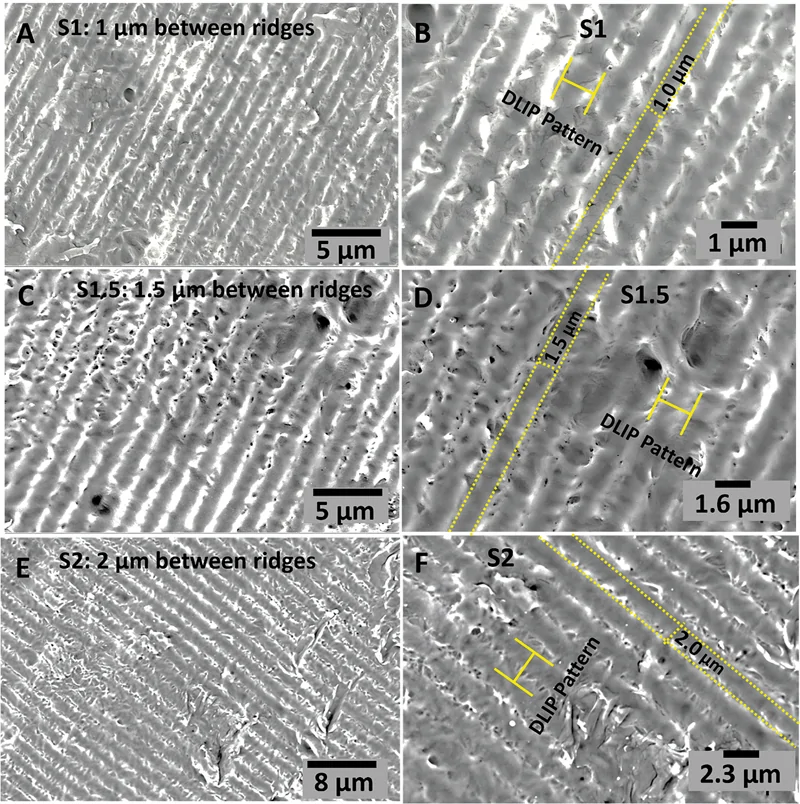
Scanning electron microscopy (SEM) images ( **A** & **B** ), ( **C** & **D** ), and ( **E** & **F** ) of S1, S1.5, and S2 of the surface of polyether-ether-ketone (PEEK) coating functionalized by direct laser interference patterning (DLIP) created periodic patterns with different spatial periods of 1, 1.5, and 2 μm, respectively.

### Contact Angle

Contact angles of DLIP fabricated patterns on PEEK coating with spatial periods of 1, 1.5, and 2.0 µm on S1, S1.5, and S2, respectively. PEEK coating is hydrophobic because of the presence of a nonpolar aromatic ring and the weak intermolecular forces at the surface of PEEK coating deposited on 316L SS. Both factors minimized the interaction with polar molecules of water, and these water molecules tended to bead up on the surface of the coating and increased the value of the contact angle and lowered the wettability. Contact angle values of PEEK coating increased and wettability decreased when the surface of PEEK was functionalized by DLIP, which created line-like textures with spatial periods of 1, 1.5, and 2 µm on S1, S1.5, and S2, respectively, as shown in the SEM image ( Fig. 2A–F ). Fig. 3 shows the comparison among the measured values of contact angles of S0, S1, S1.5, and S2 and reported with standard deviation, where *n* = 3 for the validity of data.

**Fig. 3:**
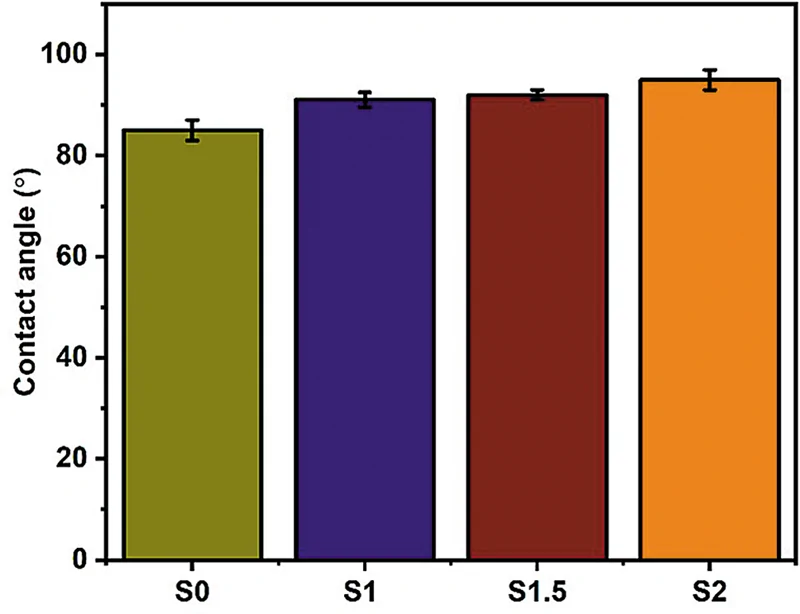
Shows the comparison among the values of the contact angle of S0, S1, S1.5, and S2.

The wettability of PEEK coating after creating the patterns by DLIP was reduced due to increasing the contact angle values from 85 ± 2 degrees to 91 ± 1.5 degrees, 92 ± 1 degrees, and 95 ± 2 degrees by increasing the spatial periods from 0 to 1 µm, 1.5 µm, and 2.0 µm. It was assumed that the air was entrapped beneath the water droplet, which minimized the liquid and liquid contact as the separation between the ridges of patterns increased, which ultimately increased the hydrophobic effect causing increase in the values of contact angles according to the Cassie–Baxter wetting regime.

### Surface Roughness

The values of average surface roughness of PEEK coating and DLIP-functionalized PEEK coating having patterns with spatial periods of 1, 1.5, and 2 µm on S1, S1.5, and S2, respectively, were measured by a surface profilometer. PEEK coating exhibited average surface roughness of 1.90 ± 0.04 µm, but the values of average surface roughness were increased from 1.90 ± 0.04 to 1.96 ± 0.02 µm, 2.04 ± 0.03 µm, and 2.10 ± 0.04 µm and the separation between ridges of patterns increased from 0 to 1 µm, 1.5 µm, and 2.0 µm, respectively. Fig. 4 shows the comparison of average surface roughness values measured by the stylus profilometer of the surface of S0, S1, S1.5, and S2.

**Fig. 4:**
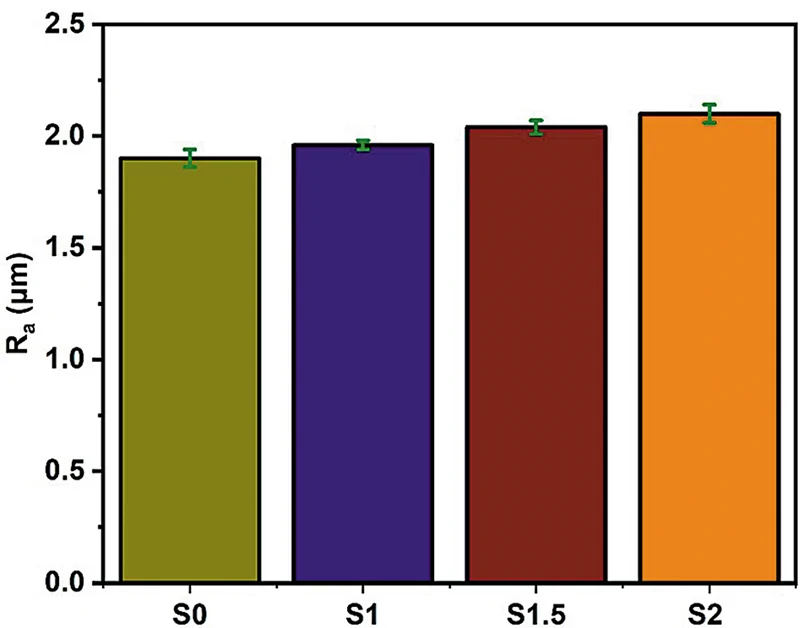
Shows the comparison among the values of average surface roughness measured by the stylus profilometer of the S0, S1, S1.5, and S2.

This increase in average surface roughness might be due to DLIP, which functionalized the structure of PEEK coating by creating patterns having ridges and grooves. These ridges and grooves caused to increase in the average surface roughness of DLIP-functionalized PEEK coating. The cellular attachment with the surface does not depend on only one factor, which is the contact angle, because other factors such as surface roughness and surface topography/surface chemistry also play an important role in the cellular attachment. Virk et al 35 reported the average surface roughness of 1.8 ± 0.1 µm of PEEK/curcumin/hBN composite coating deposited on 316L SS. Atiq Ur Rehman et al 36 and Ureña et al 37 reported that 1 to 2 µm value of average surface roughness is ideal for cellular response of surface. The value of average surface roughness is reported in this study lies in the range of the value of surface roughness required for cellular attachment.

### Antibacterial Analysis

PEEK coating deposited on 316L SS via EPD was further functionalized by DLIP, which created patterns with a spatial period of 1, 1.5, and 2 µm on S1, S1.5, and S2, respectively. These DLIP-functionalized PEEK-coated samples were subjected to turbidity against Gram-negative strain ( *E. coli* ) test for 24 hours of incubation time. Fig. 5 shows OD _600_ of the control sample unfunctionalized PEEK coating remained greater than 0.015 but below 0.020 after incubation of 6 hours, but its value increased to 0.080 after 12 hours. This indicated that the growth of *E. coli* increased on the unfunctionalized surface of the PEEK coating. The growth of *E. coli* on the functionalized surface of PEEK having a 1-µm spatial period between ridges showed that the growth of *E. coli* increased from 0.020 to 0.040 after 24 hours of incubation, which was indicated by increasing OD _600_ .

**Fig. 5:**
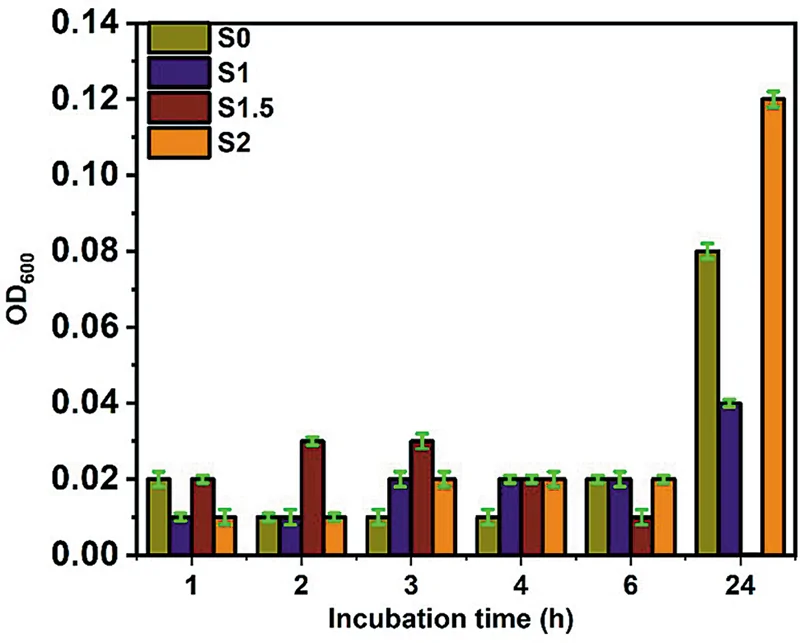
The comparison of ODs direct laser interference patterning (DLIP) functionalized surface of polyether-ether-ketone (PEEK) coating of S1, S1.5, S2 and unfunctionalized surface of PEEK coating by turbidity test after 24 hours of incubation, and the average values of OD of each sample were reported with a standard deviation ( *n* = 3).

DLIP-functionalized PEEK coating sample, which had a 1.5-µm spatial period between ridges of periodic patterns, showed that *E. coli* growth increased as indicated by an increase in OD _600_ from 0.020 to 0.030 after 3 hours of incubation but its value decreased to 0.020 after 4 hours of incubation and further decreased to 0.015 after 6 hours and finally reached to zero after 24 hours of incubation. This observation indicated that *E. coli* growth increased in the beginning of up to 3 hours then it started to decrease and finally reached zero concentration of *E. coli* on the DLIP-functionalized surface of the PEEK coating sample having a 1.5 spatial period between the ridges. The DLIP-functionalized surface of PEEK coating whose pattern had a 2-µm spatial period between the ridges also showed almost similar behavior for the growth of *E. coli* because the OD _600_ increased from 0.015 (control) and reached 0.12 after 24 hours of incubation, which indicated that the surface was favorable for the growth of *E. coli* .

### Cell Viability

The viability of S0, S1, S1.5, and S2 toward MG-63 cells was evaluated by comparing them with bare cultured MG-63 cells referred to as TCP. As evident from Fig. 6 , S1.5 appeared to be cell viable in comparison to S0, S1, and S2. The viability of surface functionalized S1, S1.5, and S2 was significantly higher than that of TCP and S0.

**Fig. 6:**
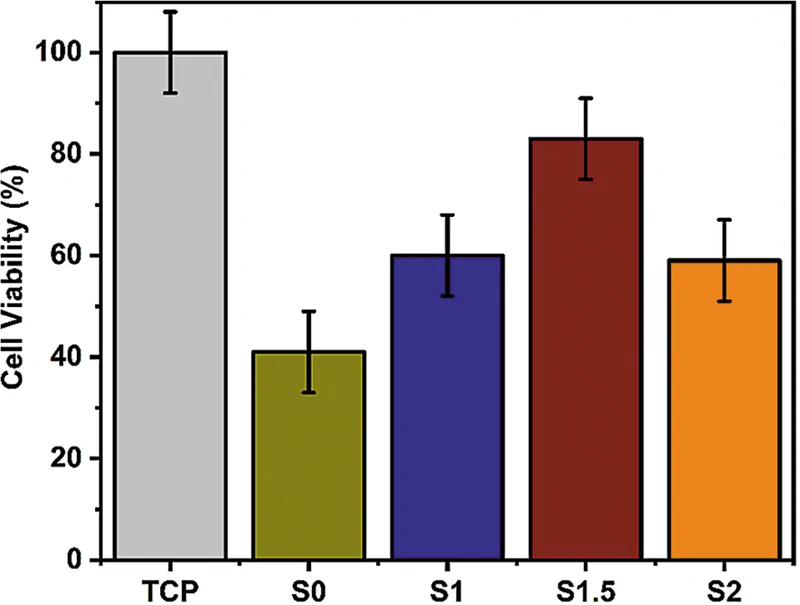
Cell viability of direct laser interference patterning (DLIP)-functionalized polyether-ether-ketone (PEEK) coating deposited on 316L stainless steel (SS) having patterns with different periodic spatial compared with tissue culture polystyrene (TCP) and unfunctionalized PEEK coating sample.

The highest viability was seen for S1.5 that makes it optimal for cellular attachment and proliferation. This may be due to the optimal roughness created via 1.5 µm patterns, such patterns may match the size of MG-63 filopodia resulting in better adherence and proliferation. 38 The lesser attachment on S1 and S2 may be attributed to more confined space and coarse space, respectively.

## Discussion

The biomechanical properties of 316L SS that make it suitable for biomedical and dental implant applications include its excellent corrosion resistance, biocompatibility, and mechanical strength. The current study reported that the surface modification of 316L SS implants is made by depositing coatings via EPD, which is a facile technique to deposit different kinds of polymers, bioactive glass nanoparticles, and natural herbs. 2 PEEK is a versatile synthetic biopolymer whose elastic modulus (3–5 GPa) is close to that of the cortical bone (7–30 GPa). 3 It is insoluble in water but soluble in sulfuric acid and exhibits good wear and corrosion resistance in physiological fluids. 39 PEEK has been used as a coating material on 316L SS implants via EPD to improve its wear and corrosion properties. Earlier studies demonstrated that different kinds of PEEK-based antibacterial and corrosion-resistant coatings were deposited on 316L SS, which inhibited the growth of bacteria and improved the corrosion resistance. 21 22

Abdulkareem et al 40 deposited chitosan/PEEK/HA composite coating on 316L SS via EPD and investigated its antibacterial effect against *S. aureus* and *E. coli* .

Atiq et al 41 reported gentamycin-loaded chitosan/PEEK/BG particles on 316L SS composite coating and investigated the antibacterial effect of gentamycin against *S. aureus* and *E. coli* strains and also studied the PEEK/BG particles composite coating to confirm the antibacterial effect of coating deposited on 316L SS via EPD. Similarly, chitosan/PEEK/Ag-Mn mesoporous bioactive glass nanoparticles (MBGNs) composite coating on 316L SS was investigated for the antimicrobial effect of the coating due to Ag metallic ions doped in MBGNs against *E. coli* , and *S. carnosus* . 21 Nawaz et al 22 deposited multifunctional composite coating on 316L SS via EPD and investigated the antibacterial effect against *S. carnosus* and *E. coli* . Virk et al 42 investigated the antibacterial effect against *S. aureus* and *E. coli* of curcumin-loaded PEEK/BG particles composite coating deposited on 316L SS. Seuss et al 43 developed an antibacterial, bioactive composite coating composed of PEEK/MBGNs and silver nanoparticles via EPD and investigated the antibacterial effect.

Several studies demonstrated that biofilm formation was inhibited by incorporating antibacterial agents like metallic ions doped in MBGNs or natural herbs in PEEK-based composite coating. 18 22 43 44 45 46 Surface modification of 316L SS by depositing a coating improves the overall surface properties of 316L SS implant, but these properties can also be functionalized by the DLIP technique, which is a robust technique for texturing coatings. DLIP method is based on the local surface modification process, which creates periodic surface patterns with adjustable pitch and geometry when two or more coherent laser beams interfere with the surface of a material at a micro- and submicron level to play a vital role in modifying its properties. 25 47

DLIP functionalized surfaces of PEEK coating deposited on 316L SS and these patterns were created by DLIP with spatial periods of 1, 1.5, and 2.0 µm on the surfaces of S1, S1.5, and S2. 48 The topographical analysis confirmed that the ridges and grooves of the pattern on S1 exhibited nonuniform and very rough topography. These ridges were separated by 1 µm as a spatial period. This rough and nonuniform surface of the ridges also indicated the localized coating material ablation or there was insufficient PEEK coating integrity along the patterned lines at certain areas of the ridges.

The DLIP-functionalized surface of PEEK coating of 1.5 µm spatial period of ridges of patterns inhibited the *E. coli* growth after 24 hours of incubation, which indicated that such a surface is favorable for implant design with optimal antibacterial surface. This antibacterial effect induced in the functionalized surface could be due to the following factors. The specific spatial period of 1.5 µm of ridges of pattern created by DLIP on PEEK coating might be a perfect size to physically interfere with *E. coli* with the surface and support the initial adhesion but prevent a stable attachment of *E. coli* . This physical disruption could induce mechanical stress on the bacterial cells of *E. coli* and lead to damage or rupture of the cell membrane, as a result the growth of *E. coli* was inhibited, which was demonstrated by a decrease in optical density after 24 hours of incubation. Second, a specific topography of the functionalized surface of PEEK coating exhibiting a 1.5-µm spatial period also inhibited the quorum sensing of *E. coli* for biofilm formation by accumulating the critical mass of bacterial cells. Without initial stable bacterial attachment which leads to the biofilm formation on the surface, bacterial growth and proliferation became impossible which resulted in *E. coli* bacteria dying as demonstrated by zero optical density after 24 hours of incubation. Fig. 6 shows the cytocompatibility of the functionalized surface of PEEK deposited on 316L SS of S1, S1.5, and S2 samples compared with nonpatterned (control sample) and TCP. The cell viability on the DLIP-functionalized PEEK coating surfaces is higher than that of the nonpatterned 316L SS. The moderate roughness of S1.5 is expected to provide suitable microstructure for cellular interactions; cell attachment compared with excessively smoother (S0) and rough (S2) surface. 38 The ridge of spacing created by spatial period of 1.5 µm lead to suitable microtopography for MG-63 attachment as it led to better dimension for filopodia. 49 This led to enhanced attachment as MG-63 cells can ideally anchor with their filopodia in grooves of surface making the connection more stable and favorable. As S1.5 portrayed moderate contact angle of 92 ± 1 degrees and such surfaces being not more hydrophilic or hydrophobic promote cell attachment as such surfaces do not tend to cause excessive dehydration or limit protein absorption. 50 Moreover, the patterned surface of S1.5 provides a larger surface area for interaction of cells and better contact between 316L SS and MG-63 cells. The increased surface area can also promote signaling pathways involved in cellular ingrowth and proliferation. 51 The topographical dimensions of S1.5 seems to be well suited for biomechanical properties of MG-63 cells as they may trigger integrin-mediated pathways involved in cell survival, ultimately portraying higher viability. 52 The interplay between surface texture and cell membrane seems to be pivotal in signaling pathways resulting in better adhesion and proliferation of MG-63 cells. 52 However, the 1.5 µm topography showed suitable cell viability and may promote protein adsorption, enhancing integrin-mediated cell signaling.

## Conclusion

The present study focused on the functionalized surface of PEEK coating samples that exhibited patterns with different spatial periods of 1, 1.5, and 2 µm. The topography of PEEK coating deposited on 316L SS was successfully functionalized by DLIP which produced a regular pattern structure having ridges and grooves over a 1 × 1 cm ^2^ region with spatial periods of 1, 1.5, and 2 µm. However, compared with the unfunctionalized S0 and DLIP-functionalized surfaces of PEEK coating having patterns of spatial periods of 1 and 2 µm, it was revealed that the tailored surface of PEEK coating with a spatial period of 1.5 µm significantly reduced *E. coli* attachment on the functionalized surface of PEEK coating. The functionalized surface of PEEK coating by DLIP, having the patterns with a spatial period of 1.5 µm exhibited an average surface roughness of 2.04 ± 0.03 µm with a contact angle of 92 ± 1 degrees was a favorable surface that inhibited bacterial growth after 6 hours of incubation and reached to zero after 24 hours of incubation. Furthermore, cell viability analysis demonstrated that the DLIP-functionalized PEEK coating having a pattern with a spatial period of 1.5 µm showed 80% cellular growth as compared with the control sample, which evinced the biocompatibility for MG–63 osteoblast cells. These results demonstrate that DLIP can be used to produce passive antibacterial surfaces that can inhibit bacterial growth without incorporating any kind of drug, antimicrobial agent, or natural herb in the PEEK coating to make it a biocompatible and antibacterial surface.
